# Notes and News

**Published:** 1894-05-19

**Authors:** 


### NOTES AND NEWS.

COLLECTED AND COMMUNICATED.

### MEETINGS OF THE WEEK.

Hospital for Diseases of the Nervous System, Regent's Park, Annual General Meeting, Cavendish Rooms, Mortimer Street, Monday, May 21st, 3.0 p.m.

Hospital for Sick Children, Great Ormond Street, Annual Court, Wednesday, May 23rd, 4.30 p.m.

Royal Eye Hospital, Southwark, Festival Dinner and Reception by the Lady Mayoress, Grafton Galleries, Friday, May 25th.

---

IT has been decided to establish a Museum of Hygiene at Havre, the nucleus of the necessary funds being the surplus money from the receipts of the exhibition held at Havre. The "Progrès Médical" expresses the hope that Paris will follow the example of Havre, and establish a like institution.

---

THE Secretary of the Victoria Hospital for Children is issuing an appeal in a most attractive form. The theme of a children's hospital is always of interest. Captain Blount has made the little people who enter his institution tell their own story. Three summer scenes tell the tale of three striking contrasts. The wretched children of the slums; their playground the gutter firstly. The carefully-tended little inhabitant of the cosy cot in the hospital comes next; and, lastly happy children play as they never played before on the glorious sands at Broadstairs. Who can permit a cot to remain empty, whilst suffering little ones lie neglected in their wretched homes, and whose convalescence is spent not in building castles on the seashore, but in the poisonous atmosphere of crowded homes or in the crowded street. The convalescent home contains 50 beds. Last year the deficit in its accounts was £604. Will it be possible to send the 650 children to its hospitable shelter if this want continues?

THE overcrowding of the Paris dissecting-room continues to excite much comment. This year no less than 1,200 medical students were entitled to dissect in the very confined space contained in the rooms of the Paris Medical Faculty.

---

RATHER an amusing report of the misfortunes of Chicago appears in an American contemporary. It runs, "Chicago has had typhoid fever, the World's Fair, and W. T. Stead, and now it is suffering from small-pox!"

---

BACTERIOLOGICAL and chemical laboratories have been opened in connection with the Clinical School of the Royal Southern Hospital at Liverpool, which school shows an evident desire to be in the foremost ranks of medical progress.

---

THE Medical Battery Company has received permission to carry on the business for two months longer in order to realise assets. It is interesting to know that 86,000 appliances have been sold since the establishment of the business. Where will the credulous public now turn for an unfailing and universal panacea of all ills!

---

IT is quite a mistake to imagine that residence as patient in a hospital has not an attraction to some. The *Evening Standard* gives the case of a Frenchman who not only simulated illness so well as to be an accepted patient, but he was ultimately sent to Lourdes, with the hope that the miraculous waters of the Sacred Pool would effect the cure that secular skill had failed to accomplish. He ultimately resorted to a lunatic asylum and another hospital, when at last the fraud was exposed.

---

THE Secretary of the British Medical Association has issued the following notice for the encouragement of scientific and medical research: "The Council of the British Medical Association are prepared to receive applications for one of three Research Scholarships which is vacant, of the value of £150 per annum, tenable for one year, and subject to renewal by the Council for another year. Applications to be sent in writing addressed to the General Secretary on or before June 15th, stating the particulars of the intended research, qualifications, and work done."

---

THE cause of insomnia has ever been an interesting problem to mankind. An American brother regards cats as such important factors in the matter, that he has brought the subject down to figures. He estimates, and he considers he is far from exaggeration when doing so, that 10 per cent of the dwellers in cities have their night's rest spoilt by the howlings of cats. This is a serious accusation, and one which should make us really consider whether we are justified in adding to our own and others sleepless nights and canot find "certain death" for rats and mice other than through the agency of the domestic cat.

